# Buffalo Academy of Medicine

**Published:** 1902-03

**Authors:** Irving P. Lyon

**Affiliations:** Secretary


### SOCIETY PROCEEDINGS.

### Buffalo Academy of Medicine.

*Section on Pathology, January 21, 1902.*

Reported by IRVING P. LYON, M. D., Secretary.

The regular monthly meeting of the Buffalo Academy of Medicine was held in the Academy rooms, Public Library Building, Tuesday, January 21, 1902, at 8.30 p.m., Dr. Grover W. Wende, chairman, presiding.

Attendance, 22. The minutes of the previous meeting were read and approved.

Dr. Herman E. Hayd presented and discussed the following pathological specimens: (1) hypertrophied prostate, middle lobe —removed by perineal section; (2) acutely inflamed appendix (appendicitis); (3) normal appendix, containing at its tip a muskmellon seed.

Dr. G. Frank Lydston, of Chicago, upon the invitation of the Academy, discussed the operative treatment of prostatic hypertrophy. Dr. Lydston declared his disapproval of the Bottini operation, as compared with radical measures, *i. e.*, prostatectomy. The Bottini operation is merely palliative, and its general employment is calculated to retard the legitimate development by radical prostatic surgery. The general practitioner should realise that prostatic hypertrophy is not strictly a disease of old men, but merely reaches its full development with symptoms demanding treatment in those of advanced years. The disease develops gradually throughout adult life and should not be permitted by the neglect or ignorance of the physician to reach a stage so advanced as to cause serious bladder symptoms. Let the diagnosis be made early and give the surgeon the call at once and the result of radical prostatic surgery will be comparable to those obtained in other early operations. The general practitioner must be educated to this view of prostatic disease.

In reply to questions from Dr. Lyon, Dr. Hayd stated that the patient whose appendix contained a muskmellon seed had presented none of the usual symptoms of appendicitis. Loss of weight and occasional pain in the abdomen were the only symptoms and seemed to justify an exploratory operation.

The first paper of the evening was read by Dr. G. Frank Lydston, of Chicago, on

THE RELATION OF EVOLUTION TO INFECTION, WITH SPECIAL REFERENCE TO VENEREAL DISEASES, and presented in detail Dr. Lydston's well known views on the variability of pathogenic organisms and their effects, explained by their tendency to revert toward a less highly specialised form (atavism), a principle well understood by students of evolution. The general rule governing such variation may be stated in the formula: The tendency to variation of any species is in inverse ratio to its degree of specialisation by evolution. Microörganisms, being among the lowest and simplest forms of life, are little specialised, relatively, and are consequently subject to wide variations in their pathogenicity. The gonococcus is not always the same organism in its capacity to cause a specific urethritis. Indeed, the gonococcus may be found in the urethra without its having ever excited any inflammatory reaction; in other words, "the gonococcus is not always the gonococcus." The same fact is true of the diphtheria bacillus, the diplococcus of pneumonia, and numerous if not all other pathogenic microörganisms.

Every disease germ is subject to modification in its disease-producing capacity by its environment, by the suitability of the soil upon which it grows, whether in test-tube culture or in the tissue-soil of its victim. This tissue-soil in any individual may be so unfavorable, by reason of a natural immunity or resistance possessed by him, as to render the germ relatively or absolutely harmless.

We have to recognise, then, in the power of germs to produce disease, the two factors of the variability of germ-species and the resistance offered to them by unfavorable tissue-soil in the individual. The interrelation by these two factors determines the degree of infection.

This variability in the capacity of germ species to cause disease has not yet been grasped by the medical profession, though bacteriologists and pathologists are beginning to reckon with it. There is nothing novel or iconoclastic in accepting this doctrine. It is simply the application of the principles of evolution and involution to germs, and to germs these principles apply with particular force because germs are so little specialised and therefore so poorly fixed species.

Dr. Lydston's paper was discussed and its general conclusions endorsed by Drs. Park, Stockton, Blaauw, Gaylord and Dowd. A vote of thanks was passed tendering to Dr. Lydston an expression of the appreciation of the Academy for his able address.

The second paper of the meeting was read by Dr. J. HENRY DOWD, entitled,

NEW TISSUE FORMATION, ITS EARLY DETECTION AND COMPLETE OBLITERATION.

The meeting adjourned at 11.20 p.m.

